# Thromboexclusion Procedure for a Recurrent Descending Aortic Pseudoaneurysm in the Pre-Stent Era

**DOI:** 10.1016/j.atssr.2025.07.015

**Published:** 2025-08-15

**Authors:** Sebastien Strachan, Mohammad A. Zafar, Bulat A. Ziganshin, John A. Elefteriades

**Affiliations:** 1 Aortic Institute at Yale-New Haven Hospital, Yale University School of Medicine, New Haven, Connecticut

## Abstract

We present a dramatic case of coarctation of the descending aorta. Standard surgical treatment led to graft infection, necessitating a complex series of additional surgical procedures—all without resolution. A classical “thromboexclusion” procedure accomplished durable, decades-long survival. The thromboexclusion produced the expected thrombotic occlusion of the descending aorta. The extra-anatomic ascending-to-abdominal bypass graft provided long-term blood flow to the abdominal organs and lower extremities.

Aortic pseudoaneurysms carry a high rupture risk, particularly in patients with multiple prior aortic operations. Before the introduction of endovascular repair, open surgical techniques, including thromboexclusion, were used to manage complex aortic pathologies, including aneurysms and dissections. Thromboexclusion was used specifically to defunctionalize descending pathologies without cardiopulmonary bypass and direct graft replacement.

The concept of thromboexclusion, developed by Carpentier and colleagues,^1^ involves using an extra-anatomic bypass graft to divert blood flow from the ascending to the abdominal aorta (Figure). This induces flow reversal in the descending aorta, causing blood stagnation and “therapeutic” thrombosis, thereby reducing the risk of rupture of any descending aortic pathology and allowing spinal cord adaptation.^2^^,^^3^ Perfusion to vital branches is maintained.^2^

**Figure:**
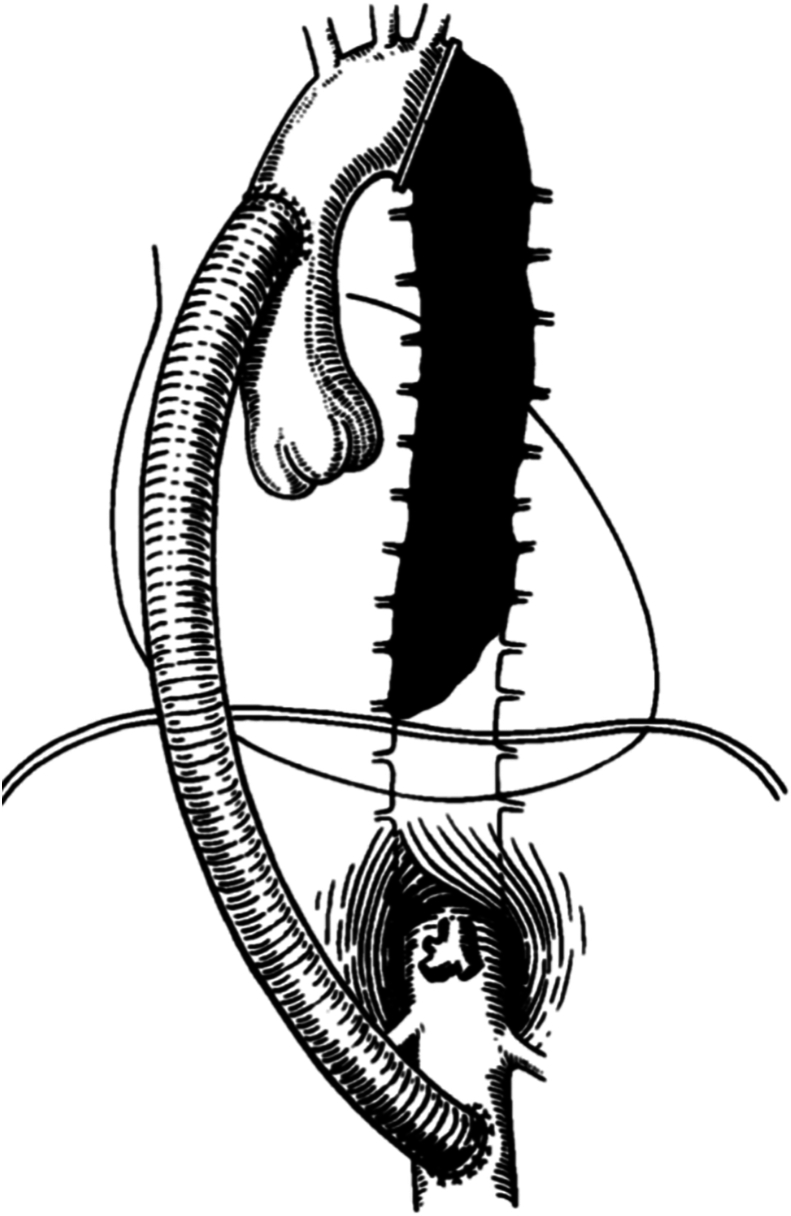
Representation of the thromboexclusion operation. Reproduced from Elefteriades and colleagues^2^ with permission from The Society of Thoracic Surgeons.

Thromboexclusion offers advantages, including performance on nonpathologic segments, reduced invasiveness, and lower paraplegia risk due to uninterrupted lower-body blood flow.^1^, ^2^, ^3^ The graft is constructed and opened to blood flow before the descending aorta is permanently clamped. At Yale University, in that era, we used a metal, serrated clamp of our own construction.

Thromboexclusion carries risks such as rupture, potential clamp migration, and retrograde dissection (to the ascending aorta).^2^ Some experts felt the surgical procedure of thromboexclusion was complex enough that it may not significantly reduce mortality rates compared with more traditional techniques. Advances for aortic aneurysm repair, including graft replacement and endovascular repair stent grafts, have largely replaced thromboexclusion, making this a rarely used approach today.

There are limited reports of long-term follow-up outcomes in survivors of the thromboexclusion procedure. Here, we present a 22-year follow-up of a patient with a complex thoracic aortic history who underwent extra-anatomic bypass and thromboexclusion for a recurrent descending aortic pseudoaneurysm.

A 50-year-old man with a history of complex aortic pathology presented to our team in 2003 for elective management of a persistent descending thoracic aortic pseudoaneurysm with continued flow. His medical history was significant for coarctation of the aorta diagnosed at age 15 after a chest roentgenogram for a football-related rib fracture, surgically repaired in 1968. Likely due to an undiagnosed bicuspid valve, ascending aortic/root dilation later developed, requiring a Bentall procedure in 1988.

In 1999, he experienced acute hemoptysis from a ruptured descending aortic pseudoaneurysm at his prior coarctation repair site, resulting in massive hemorrhage into the left upper lobe. Emergent thoracotomy, partial left upper lobectomy, and aneurysm repair were performed. His postoperative course was complicated by acute respiratory distress syndrome, requiring prolonged intubation, followed by left vocal cord paralysis requiring a prosthesis.

Given his history of multiple prior thoracic operations and the complexity of his aortic pathology, among the management options for his persistent pseudoaneurysm we selected, in that era, a thromboexclusion procedure.

### Surgical Procedure

The left subclavian artery was exposed. Median sternotomy, midline laparotomy, and redo sternotomy were performed, with lysis of substernal adhesions to the heart.

Preoperative angiography suggested close proximity of the left common carotid and subclavian artery origins. Intraoperatively, this was confirmed, and placing the thromboexclusion clamp proximal to the left common carotid artery was determined safest. The old ascending aneurysm repair was exposed, revealing prior sutures and a wrapped aneurysmal shell around the Bentall graft. The innominate, left common carotid, and left subclavian arteries were dissected, with the vagus and phrenic nerves protected. Simultaneously, the infrarenal abdominal aorta was exposed.

Limited exposure and tissue safety concerns at the aortic arch prompted proximal anastomosis to the innominate artery using an 18-mm Hemashield graft (Boston Scientific Corp), constructed from two 30-mm Hemashield segments sewn together with 4-0 Prolene (Ethicon) and felt reinforcement. The proximal anastomosis was performed on the anteroinferior surface of the innominate artery, which appeared slightly enlarged.

The graft was routed through the transverse mesocolon, base of the small bowel mesentery, and into the infrarenal aortic position. The distal anastomosis was performed in an end-to-side fashion at the level of the inferior mesenteric artery using continuous 3-0 Prolene. After clamp removal, hemostasis was confirmed, with a strong pulse in the graft and distal aorta.

Attention was then redirected to the chest, where the left common carotid artery was further mobilized and transposed to the innominate artery. The left carotid aortic stump was closed with a running Prolene suture, reinforced with mattress sutures. The proximal left subclavian artery was doubly ligated and secured with large urology clips.

Next, an 8-mm externally reinforced polytetrafluoroethylene shunt graft was anastomosed between the newly constructed innominate-to-infrarenal aortic bypass graft and the left subclavian artery in an end-to-side fashion using Prolene sutures.

After air was removed from the 18-mm Hemashield graft, a permanent aortic clamp was placed proximal to the left common carotid artery stump under lowered systolic blood pressure.

Intraoperative echocardiography confirmed the expected rapid thrombus formation in the upper descending aorta.

### Outcome and Follow-Up

In 2007, 4 years postoperatively, follow-up CT imaging revealed persistent forward leakage through the permanent aortic occlusion clamp and progressive aneurysm enlargement. Given these findings, the patient underwent cardiac catheterization, during which 2 Amplatzer devices (St. Jude Medical) were deployed to seal the leakage sites.

Follow-up CT imaging confirmed successful cessation of the leak, complete thrombosis of the descending thoracic aorta, and stability of the aneurysm, with no significant change in size compared with previous examinations.

Recently, we were surprised to find the patient doing perfectly 22 years after his thromboexclusion procedure. He has remained in excellent clinical condition. Serial CT angiography has demonstrated stable thrombosis of the excluded descending thoracic aorta without evidence of further aneurysmal progression. The extra-anatomic descending aortic bypass graft remains widely patent, and the reimplanted innominate, left common carotid, and left subclavian artery grafts have maintained long-term patency, with no signs of stenosis.

## Comment

This case illustrates the long-term outcomes and durability of thromboexclusion as a surgical technique for managing complex aortic pathology, particularly in the prestent era. The patient underwent a challenging but ultimately successful extra-anatomic bypass and thromboexclusion for a recurrent descending aortic pseudoaneurysm, achieving excellent clinical stability over a 22-year follow-up period. See Supplemental Video S1, which scrolls through the sagittal plane of the CT angiography chest and abdomen.

The Carpentier thromboexclusion offered an alternative to traditional open descending aortic replacement in cases where direct graft placement was high-risk or contraindicated.^1^ In this patient, with multiple thoracic operations and complex pathology, the procedure treated a recurrent pseudoaneurysm with persistent flow and imminent rupture risk. The surgical site was clearly inimitable for direct approach.

One major concern with thromboexclusion is the potential incomplete thrombosis of the excluded descending thoracic aorta, leading to persistent flow and continued aneurysmal progression. This is not commonly seen. Our “homegrown” occlusion clamp worked well in many cases, but here, the severe inflammation, fibrosis, and copious foreign material (including bovine pericardium) prevented complete sealing, requiring Amplatzer plugs 4 years postoperatively. Therefore, although thromboexclusion provides an immediate solution aneurysm exclusion, long-term surveillance is essential to detect persistent perfusion or enlargement.

Despite these challenges, our case demonstrates that thromboexclusion can achieve long-term success with appropriate patient selection, surgical technique, and diligent follow-up. The patient remained clinically stable, with serial imaging confirming the stability of the excluded descending thoracic aorta and patency of the extra-anatomic bypass. Additionally, the aortic branch grafts for the left common carotid and left subclavian arteries remained widely patent, demonstrating the efficacy of the initial revascularization strategy.

This case highlights the importance of an individualized surgical strategy for aortic pathology, particularly after multiple aortic procedures. Although modern endovascular techniques have largely replaced thromboexclusion, understanding the principles behind this historical surgical approach remains valuable. In fact, thromboexclusion could have application even in the current era, in select cases where endovascular repair is not feasible or is contraindicated. In the earlier era, we applied thromboexclusion as a definitive treatment for descending aortic dissection.
